# Notes

**Published:** 1896-03

**Authors:** 


### Notes.

Dr. C. M. Drake, chief surgeon Southern Railway has moved his headquarters from Knoxville to Atlanta.

Dr. Dunbar Roy has been appointed consulting ophthalmologist to the Western Division of the Southern Railway.

Dr. C. D. Hurt has been appointed physician to the Grady Hospital to fill the vacancy occasioned by the resignation of Dr. J. S. Todd.

Dr. S. C. Courtney Pinkney, late of New York, has taken up his residence permanently in Atlanta. Dr. Pinkney will limit his practice to the diseases of the mind and nervous system, with which he has had a large experience in the clinics of New York.

Dr. A. H. Van Dyke, formerly house surgeon of the Hospital for Ruptured and Crippled, New York, has returned to Atlanta and will resume practice, giving especial attention to orthopedic surgery.

Dr. A. L. Harris, for many years a prominent citizen of Atlanta, died in January. Dr. Harris was but a recent graduate in medicine, having been, all his life, identified with newspaper work.

The commission appointed to examine into the sanity of Alexander Carr, the assassin of Capt. H. A. King, have reported that Carr is suffering from dementia and is, therefore, irresponsible. Dr. James B. Baird, one of the members of the commission, dissented from the above opinion and gives it as his belief that the prisoner is a simulant, but that there is some ground for reasonable doubt. There is no doubt, however, in the mind of the people, but that Carr was sane at the time the crime was committed, whatever be his mental condition now.

DR. C. S. WEBB, who was forced to resign the chair of medicine at the Southern Medical College on account of ill health, has returned to his native home in Virginia. Our good wishes and hopes for a speedy restoration to health go with him.

---

DR. W. S. ARMSTRONG died suddenly while attending a meeting of the Medical Board of the Grady Hospital on February 11. Dr. Armstrong had been in poor health for some time, but a fatal issue was unexpected. The deceased was one of the best known surgeons and teachers in Georgia. He was, for years, professor of Anatomy and Clinical Surgery in the Atlanta Medical College which chair he filled with distinguished ability. He was also attending surgeon to the Grady Hospital. Dr. Armstrong served through the war as surgeon in the Confederate army. He was a clear and forceful teacher and a skillful anatomist. He enjoyed, in a marked degree, the regard and respect of his colleagues in the profession and was beloved of the people for his charity and largeness of heart. He was a surgeon of a high order of ability and large experience.

Dr. Armstrong leaves a widow and two children to mourn his loss, a daughter and a son; the latter a student at the College of Physicians and Surgeons, New York. Resolutions commemorative of the respect and esteem in which he was held have been drawn up by the Medical Board of the Grady Hospital and the faculty and students of the Atlanta Medical College.

---

THE following, is the report of the committee appointed by the Atlanta Medical Society upon the death of Dr. W. S. Armstrong:

HALL OF THE ATLANTA SOCIETY OF MEDICINE.

Pursuant to the appointment of the president, your committee respectfully submits the following report touching the recent death of our fellow-member, Dr. William Simpson Armstrong:

Dr. Armstrong died suddenly Tuesday evening, February 11, 1896, in the reception room of the Grady Hospital, at which place he had arrived only a few minutes before for the purpose of attending a regular meeting of the Medical Board, to the presidency of which he was recently elected.

Dr. Armstrong was born in Washington, Wilkes county, Georgia, in 1838. After a liberal preliminary education, he commenced the study of medicine under Dr J. H. Lane, of Washington, and after attending one course of lectures in Augusta, he matriculated in the medical department of the University of New York and graduated from that institution in 1859.

He served with credit to himself as surgeon in the Confederate army from 1861 to 1865. Soon after the close of the war, he went to Europe to further prosecute his medical studies, and upon his return home, he located in Atlanta and commenced the general practice of medicine.

In 1867, he was elected to the Chair of Anatomy in the Atlanta Medical College, after having served one year as demonstrator, and he continued to fill that position with distinguished ability to the day of his death. In 1890, clinical surgery was added to the chair of anatomy.

He was one of the original members of the Surgical Staff of the Grady Hospital, and proved himself, in that capacity, an ardent and invaluable worker.

He was a member and the president of the Board of Health of the city of Atlanta for fourteen years, until he resigned in 1893; and he accomplished extremely valuable results for the sanitary interests of the city, during a very important period of its history and growth.

In the practice of his profession, he exhibited a strong predilection for surgery. His enthusiasm in his work was remarkable and contagious. His earnestness and fidelity were conspicuous and invariable. During the latter years of his life, he performed many of the most intricate and difficult of the major surgical operations, and achieved signal and brilliant results.

His excellence of character, his usefulness, and his uniform kindness and consideration, were universally recognized, and, with all of his admirable traits and all of his professional accomplishments, he was as modest and unassuming as a little child.

His whole life—public, private, religious, and professional—was free from stain. It was marked by faithfulness, honesty and truth and should serve as a shining example to those who desire to meet their obligations to their Maker and their fellow-men.

JAMES B. BAIRD, M. D.,

W. S. KENDRICK, M. D.,

Committee.

Atlanta, February 18, 1896.

